# Tackling Food Waste: An Exploratory Case Study on Consumer Behavior in Romania

**DOI:** 10.3390/foods13203313

**Published:** 2024-10-18

**Authors:** Cristina-Anca Danciu, Alin Croitoru, Iuliana Antonie, Anca Tulbure, Agatha Popescu, Cristian Stanciu, Camelia Sava, Mirela Stanciu

**Affiliations:** 1 Faculty of Agricultural Sciences, Food Industry and Environmental Protection, Lucian Blaga University of Sibiu, 7-9 Dr. Ion Ratiu Str., 550012 Sibiu, Romania; cristina.danciu@ulbsibiu.ro (C.-A.D.); iuliana.antonie@ulbsibiu.ro (I.A.); anca.tulbure@ulbsibiu.ro (A.T.); camelia.sava@ulbsibiu.ro (C.S.); 2 Faculty of Social Sciences and Humanities, Lucian Blaga University of Sibiu, 5-7 Victoriei Blvd., 550324 Sibiu, Romania; alin.croitoru@ulbsibiu.ro; 3 Faculty of Management and Rural Development, University of Agronomic Sciences and Veterinary Medicine, 59 Marasti Blvd., District 1, 011464 Bucharest, Romania; agatha_popescu@yahoo.com; 4 Faculty of Sciences, Lucian Blaga University of Sibiu, 5-7 Dr. Ion Rațiu Str., 550012 Sibiu, Romania; cristiandumitru.stanciu@ulbsibiu.ro

**Keywords:** food waste, consumer behavior, food waste avoidance, composite index for measuring food waste avoidance, Romania

## Abstract

The scourge of food waste (FW) is a significant global challenge, impacting climate change, food security, and the sustainability of agrifood systems. The objective of this paper is to identify, analyze, and understand the factors influencing household consumer behaviors in Romania regarding the reduction of FW. Three primary research objectives were established to assess food consumption behaviors within households, to explore attitudes toward FW, and to understand the motivations for reducing FW along with the measures implemented by households to address this issue. Methodology: Data were collected through an online self-administered questionnaire, designed to investigate consumer behaviors related to the avoidance of FW. A descriptive statistical analysis was performed, and a linear regression model was developed to evaluate a composite index measuring Romanian consumers’ behavior towards FW reduction. Results: The resulting model identifies key predictors that drive concrete actions to minimize FW, including the desire to mitigate the environmental impact, household conversations about FW and strategies to reduce it, established food routines, the influence of one’s social circle, individual ecological and social responsibility, and the effectiveness of awareness campaigns addressing the consequences of FW. Practical and social implications: The findings highlight the necessity of education and awareness initiatives to shift attitudes and behaviors concerning FW. Future research is warranted to deepen understanding and enhance interventions. Originality: This study represents a pioneering and innovative inquiry into FW behavior in Romania, filling a gap in the existing literature and contributing to the broader discourse on this pressing environmental issue.

## 1. Introduction

Food waste (FW) refers to food discarded by retailers, consumers, or other sectors of the food supply chain. The Food and Agriculture Organization (FAO) estimates that around 14 percent of the world’s food (valued at USD 400 billion per year) continues to be wasted after it is harvested and before it reaches the shops. At the same time, UNEP’s Food Waste Index Report shows that a further 17% of our food is wasted in retail and by consumers, particularly in households [1] (UNEP Food Waste Index Report 2021|UNEP—UN Environment Programme). The FAO estimates that the food that is wasted could feed 1.26 billion hungry people every year [2].

Statistical data published by Eurostat conclude that the level of food waste in the EU in 2021 was 58 million tons of fresh food, of which approximately 54% was generated by the household consumer sector. For the same year, the level of FW per capita reached 131 kg. Misniakiewicz et al., (2024) show that around 65% of the food waste generated in Europe comes from five countries [3]. These include Germany (10,922,321 tons), France (8,764,999 tons), Italy (8,291,265 tons), Poland (4,281,212 tons), and Spain (4,260,845 tons). At the opposite pole are Malta (79,589 tons) and Luxembourg (83,622 tons) [4]. A report published by EUFIC states that losses and waste are more carbon-intensive for processed products that encompass many additional resources accumulated along the supply chain. The carbon footprint of FW differs according to the category of food that generates food waste, with a direct impact on the environment [5].

According to the FUSIONS (Food Use for Social Innovation by Optimizing Waste Prevention Strategies), a project with 21 partners from 13 EU countries, an estimated 100 Mtons of food waste is produced each year in the EU and the FW which can be avoided represents an average economic cost of EUR 595 per household per year [6].

FW waste can occur at various stages of the supply chain due to factors such as inefficiencies in production, transportation, storage, distribution, consumer behavior, cosmetic standards, and expiration dates [7,8,9,10,11]. FW contributes to environmental degradation through greenhouse gas emissions from decomposing organic matter in landfills, deforestation, water wastage, and energy consumption associated with production and transportation [12,13]. From a social and economic point of view, FW represents a missed opportunity to alleviate hunger and food insecurity, especially in regions where food access is limited. It also translates into economic losses for producers, processors, transporters, retailers, and consumers [14,15,16].

The forecasts related to the numerical growth of the population throughout the world show that humanity will reach approximately 9.8 billion people in 2050. Numerous authors claim that halving FW in the agri-food chain by the year 2030 will meet the current and future food needs of humanity, by the United Nations’ Sustainable Development Goal 12.3 [17,18]. This reduction can only be achieved under the joint efforts of all interested stakeholders [19]. The methods used to estimate the level of food waste must be relevant, representative, and reliable [20].

At a time of rising global hunger and surging food prices, various countries have developed national strategies and action plans to address the FW issue [2,21]. Also, organizations and businesses are implementing multiple strategies to tackle FW [22,23]. These include improving storage and transportation infrastructure, enhancing supply chain efficiency through planning, promoting consumer awareness and behavior change, redistributing surplus food to those in need, preventing FW through feeding animals, and developing innovative technologies and digital apps [24] and tools [25] for food preservation and waste reduction [26,27,28,29,30,31].

In households, FW is closely related to dietary behavior acquired throughout life [17] and depends on motivational factors, skills, and opportunities as well as current personal food management practices [32,33]. FW in households is produced in three stages, starting from purchasing food, storage, and consumption [34]. All these are closely related to the category of food [35], the purchasing place [36], the production system of food, and eating habits [37]. Household food consumption habits, frequency of cooking and consumption of home-prepared meals, preferences for different places of food purchase, type of diet, and preference for food produced in different farming systems (conventional, traditional, or organic), are an integral part of a responsible food behavior with direct effects on the protection of resources, the avoidance of FW, and the minimization of the negative impact on the environment.

Consumers play a crucial role in reducing FW by making mindful purchasing decisions by buying only the necessities, properly storing and using food items, minimizing overeating, and supporting initiatives that redistribute surplus food to vulnerable populations [38,39,40,41].

**Key aspects of the research on FW in Romania**

Removing the scarcity of studies regarding FW in Romania is an important objective, considering the FW’s negative impact on the economy, the environment, and food security. In Romania, FW research is mostly coordinated by governmental institutions, non-governmental organizations, and academic research institutions. According to the report published in 2024 by UNEP regarding the Food Waste Index, Romania is the only member state that did not report data in this regard to Eurostat [42].

*National-wide studies*: These are carried out by the National Institute of Statistics (INS) or the Ministry of Agriculture and Rural Development. They aim to assess the extent and characteristics of food waste along the entire agri-food chain [43].

*Sector analyses*: Research often focuses on specific sectors, such as agricultural production, food processing, distribution, and retail, to identify vulnerabilities and opportunities to reduce FW [44].

*Multiple data sources*: Researchers use a variety of data sources, including official statistics, sector organization reports, market studies, and field surveys to gain a comprehensive picture of the FW phenomenon [45,46,47].

*Economic and social impact assessment*: Studies analyze the impact of food waste on the national economy, including financial costs for producers, processors, and consumers [48,49], as well as the social implications for food security and social inclusion [50,51].

*Identifying effective solutions and interventions*: Research proposes strategies and policies to reduce food waste, including legislative measures, technological innovations [52], education and awareness programs [53], and collaborative initiatives between different stakeholders [54,55]. Some authors show that to reduce food waste, the understanding of the social value of religion and environmental behavior [56] must also be taken into account [57]. Other authors emphasize the need to develop digital technologies that encourage sustainable eating habits, efficiency, and sustainability of the food system [58], and contribute to a national culture of avoiding FW [31].

*Monitoring and evaluation*: After interventions and policies are implemented, research continues to monitor and evaluate progress in reducing food waste and identify new challenges and opportunities [59].

*The analysis of the Romanian consumer’s behavior, regarding FW, highlights the main causes*: There has been insufficient attention related to the shelf life of food, improper storage [60], behavioral differences due to gender [61], residence [62], and income [63]. Other causes that have been reported are a lack of sustainable food strategies [64], responsible food behavior, different moral attitudes towards FW, the perception of self-control over one’s behavior [65], and the purchase of excessive quantities because of retail discounts and promotions [66]. A recently published study identified five clusters of food consumption patterns in Romanians closely related to FW [67].

This study complements the specialized literature on Romanian consumer behavior about avoiding FW. The main aims are to identify, analyze, and understand the factors that influence consumer behavior regarding the avoidance of FW, to inform policymakers, guide investment, and mobilize action to reduce FW in Romania.

The article is structured as follows: Section 1: *Introduction*, where the problem addressed is presented and reference is made to the state of knowledge on this subject internationally and also in the Romanian context; Section 2: *Materials and Methods*, in which the study design, research objectives, data about the study sample, and the methods used for statistical analysis are presented; and Section 3. *Results and discussions*, which presents the results of the descriptive statistics for both the dependent and independent variables concerning FW avoidance behavior. A composite index has been developed to facilitate an objective measurement of FW avoidance behavior among consumers. The validity of this composite index was evaluated through a stepwise linear regression analysis, which aims to elucidate the impact of various determinants on the propensity to avoid FW in Romania. Discussions also include a comparative analysis with results documented by researchers in other countries. This comparison serves to highlight the nuances of FW avoidance behavior across different cultural and socio-economic settings, thereby enriching the comprehension of the multifaceted elements influencing consumer attitudes toward FW; Section 4: *Conclusions.*

## 2. Materials and Methods

### 2.1. Study Design

The article is based on survey-type research in which an online self-administered questionnaire was used, employing a non-probability sample from Romania. The data were collected in 2024, and the total sample size was 369 consumers. They were informed about the purpose of the study and data protection, and by completing the questionnaire they agreed to participate. The questionnaire was distributed online to the authors’ knowledge network and students, and through them, to people responsible for purchasing and cooking food in their families. The study participants were 18 to over 65 years old. The thematic questionnaire was specifically constructed for this research and was based both on the specialized literature and on original research ideas designed to explore these topics in a national context where there is not yet a tradition of FW research. Most of the questions included a Lickert-type scale for registering responses and the questionnaire collected standard socio-demographic information. For data processing, we used the IBM SPSS 26 software package.

**Figure 1:**
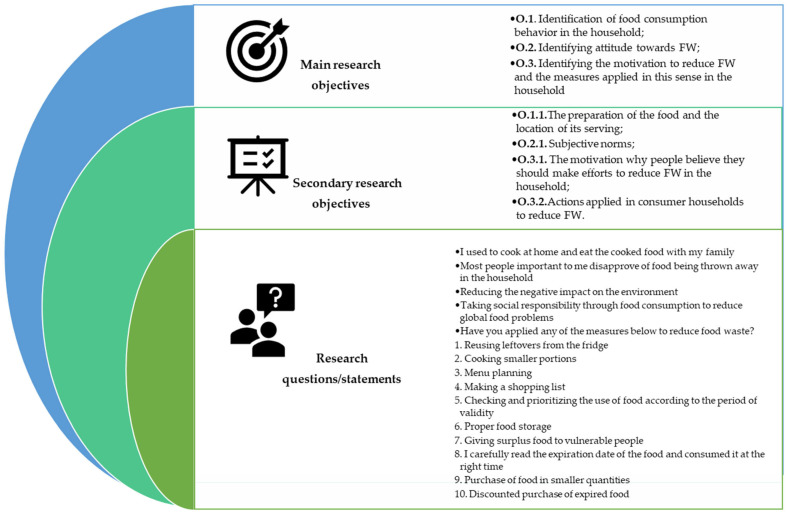
Study design. Source: Developed by the authors.

The general and specific objectives of the study are represented in Figure 1.

### 2.2. Participants

The questionnaire was completed by 376 people residing in 27 counties, out of the 41 counties and the municipality of Bucharest existing in Romania. Of these, only 369 people sent complete answers. Out of the total number of respondents, 221 people (59.89%) lived in Sibiu County. Other socio-demographic data of the sample are shown in Table 1 and Table 2.

From the above table analysis, it is shown that approximately 67% of the respondents were female, approximately 69% lived in urban areas, and approximately 76% of them were young, aged between 18 and 40. Regarding the level of education of the respondents, more than half of them were high school graduates (53.9%), and 46.01% had completed university studies (bachelor’s, master’s, and PhD). In general, as expected, the household structure included 3–4 people (55.28%). More than half of the respondents’ families (50.14%) had an average monthly income between 5001–10,000 lei (1000–2000 EUR). For 49.32% of the respondents, the monthly expenses for purchasing food necessary for the household were between 1001 and 2000 lei (200–400 EUR).

### 2.3. Statistical Analysis

The dataset allowed us to build a composite index for assessing self-declared behaviors for food waste avoidance at the individual level. This index type obtains an overall representation of a concept or construct that cannot be directly measured by a single item. The procedure of the summative index is a composite measure created by summing the individual scores of multiple items further described in this paper’s section of analyses. The composite index was also tested by employing a factor analysis, a statistical technique to uncover latent structures underlying the relationships among a set of observed variables. The primary goal of factor analysis is to reduce the dimensionality of data and identify clusters of correlated variables that can be represented by a smaller number of underlying factors.

While we employed descriptive analyses to describe and summarize the characteristics of the variables included in the analyses (mean and standard deviation), we further designed a stepwise linear regression model to test the relationships between the variance of the summative index (dependent variable) and a set of predictors explained in the section of analyses. Even though the fact that the sample is not representative of the entire population of Romania and does not allow us to generalize the conclusions, the stepwise linear regression model enabled us to test the type and the significance of the relationships between certain variables at the sample level and to draw meaningful conclusions about FW waste avoidance.

## 3. Results and Discussions

### 3.1. Descriptive Analyses

#### 3.1.1. Dependent Variable

The dependent variable was a composite index reflecting individual self-assessment regarding ten FW avoidance behaviors (Table 3). For each of these, study participants were asked to rate on a scale from 1 to 7 the frequency with which they engaged in these behaviors (where 1 represents never, and 7 always). To maintain the relevance and interpretability of the index, we opted to calculate the individual-level mean with a formula as follows: sum of individual values from i1 to i10 divided by 10 [Index = Σ (i1: i10) ÷ 10]. At the sample level, concerning the 10 indicators introduced in the analysis (Table 3), we can say that the most frequent FW avoidance behaviors were “Proper food storage” (M = 5.58) and “Reading food expiration dates carefully and consuming them on time” (M = 5.18). In contrast, the least frequent behaviors were related to “Donating excess food to vulnerable individuals” (M = 3.89). All other measured behaviors fell between these extremes of the scale and registered mean values in the range between 4 and 5.

The calculated mean scores, the standard deviation, the variance, and the coefficient of variation resulting from individual self-reports on ten food waste avoidance behaviors are shown below.

**Figure 2:**
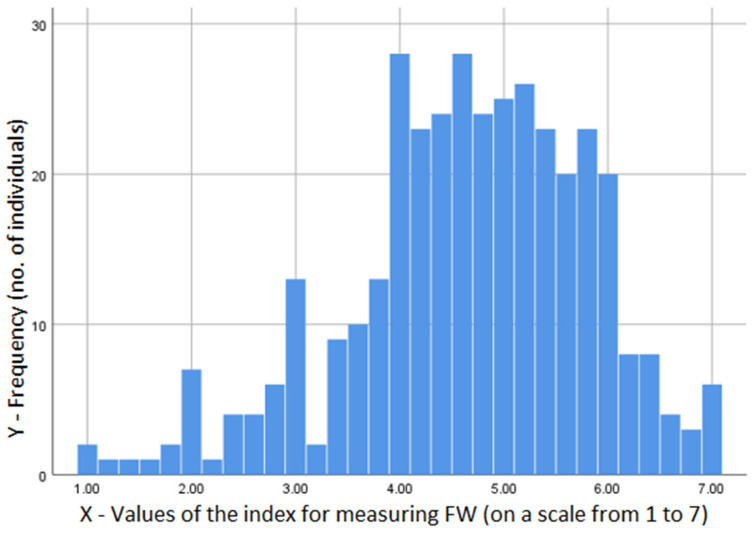
Histogram—Frequency (DV: The composite index for measuring food waste avoidance). Mean = 4.62; Std. Dev. 1.154; N = 369; (Authors’ own representation).

The coefficient of variation had values between 26.71% and 44.58%, below 35% in the case of 5 self-declared behaviors regarding the avoidance of FW. To verify if the indicators could be grouped into a one-dimensional index, we conducted a factor analysis with principal component extraction (Figure 2). This showed factors clustering on one dimension, and the Kaiser–Meyer–Olkin Measure of Sampling Adequacy (KMO) coefficient was 0.905 (the detailed results are presented in Supplementary Materials File S1). At the same time, the statistical analysis indicated excellent reliability of the instrument used, suggesting that the items were consistent and that the constructed assessment had an adequate foundation (Cronbach’s Alpha coefficient of 0.88).

#### 3.1.2. Independent Variables

To understand the variation in FW avoidance behaviors, we considered it useful to analyze the influence that a set of social values, consumption behaviors, and individual characteristics may have. In this regard, we selected the variables in Table 4 and some descriptive statistics.

The descriptive statistics revealed participants’ behaviors and attitudes towards FW reduction. The results showed a strong inclination towards cooking at home, as indicated by a high mean score of 4.30 with an SD of 0.859, signifying consistency. Motivations such as environmental impact (M = 3.93, SD = 0.969) and social responsibility (M = 3.97, SD = 1.013) were somewhat lower but still significant, exhibiting varying opinions among respondents. Participants noted perceived disapproval of FW by important individuals (M = 2.82, SD = 1.304), reflecting diverse views. Household discussions on FW (M = 0.83, SD = 0.374) and awareness of information campaigns (M = 0.62, SD = 0.487) were reported, with varying levels of exposure among respondents. The sample included more female respondents and the mean for males was 0.33 and SD of 0.470. The average age was 30.22 years (SD = 12.909), indicating diversity in age ranges. These findings provide insights into the sample specificity although the sample does not represent the entire population.

### 3.2. Linear Regression Model

Firstly, the adjusted R square values (Table 5) increased from 0.184 to 0.270 with the development of the model and this indicates that the final model (M6) explains approximately 27% of the variance in the behavior of avoiding food waste. This means that there are other factors not accounted for in the model that may also influence the behavior, but our results provide meaningful explanations regarding individuals’ behaviors in this register. From the total of IV included in the model, six factors were in statistically significant relationships with the dependent variable (DV). At the same time, gender, age, education, number of persons in the household, and household earnings were excluded from the list of variables and had no statistically significant influence within this model (their coefficients are presented in Supplementary Materials File S2). Looking at the coefficients of each IV maintained in the analysis, we can see how each variable relates to the (DV), the composite index for measuring food waste avoidance. A recently published study shows that the number of household members influences the amount of waste produced in the household, while other variables such as age, education level, or family income do not significantly influence the level of FW [68]. Other studies show that demographic features such as age and gender do not considerably influence food waste avoidance within the studied sample. The specialized literature reports the influence of consumers’ socio-demographic characteristics on sustainable food behavior [69], while food waste avoidance behavior does not depend on these characteristics [70].

Reducing the negative environmental impact (IV1) was the first and, in relative terms, the most important predictor for the index of FW avoidance within the model. It had a strong statistically significant relationship with the DV (*p* < 0.001) and a standardized coefficient 0.304. In a regression model that included multiple predictors, the fact that reducing the negative environmental impact was the most important factor indicates a very strong connection between individuals’ concerns for environmental conservation and the adjustment of their own consumption behaviors in this direction.

The second predictor included in the statistical model was related to household-level discussions about food waste and ways to reduce it (IV2). The predictor is in a positive and strong statistically significant relationship with the DV (** *p* < 0.01) and its standardized coefficient of 0.157 indicates that people who approached this issue in discussions within their household have higher values in terms of behaviors of FW waste avoidance.

This indicates that FW avoidance behaviors emerge as a result of a family environment in which the individual becomes aware of this issue and feels the need to take a stance by engaging in conversations. Encouraging discussions at the household level seems to be a direction worth considering, and in the longterm, it can have a significant spillover effect through the socialization of new generations in this spirit.

The individuals who usually cook at home (IV3) had a standardized coefficient of 0.122, indicating that this behavior correlated to food waste avoidance (*p*-value < 0.01). This result is important because it indicates how cooking at home empowers individuals and contributes to food waste reduction in Romanian society. The obtained result agrees with the one highlighted by Bobeica et al., (2024), which shows that routine and individual behavior in the household is important for reducing FW [71].

The fourth predictor included in the model was linked to the individual’s social circle. Within our sample, those who have important people in their lives disapproving of food waste (IV4) registered a negative standardized coefficient of −0.126 and the relationship was statistically significant (*p* < 0.01). Food waste avoidance behaviors have a negative relationship with the social circle in which individuals live. This can indicate that those who exhibit such behaviors perceive themselves more as exceptions about important people in their lives. This result may seem counterintuitive, but it shows that in Romania, there is not yet a culture of food waste avoidance. This may be the result, as other authors show, that most people expect actual measures to avoid food waste to be taken on different levels [71].

In a similar fashion to reducing the negative environmental impact, people motivated to shape their behaviors by taking social responsibility through food consumption to alleviate global food issues (IV5) registered higher values of the FW index. It was a positive coefficient of 0.170 and the relationship was statistically significant at *p*-value < 0.05. This suggests that individuals who are environmentally and socially responsible are more inclined to exhibit behaviors of avoiding food waste.

Last but not least, individuals’ awareness of information campaigns about food waste (IV6) had a standardized coefficient of 0.102, which means that individuals who were exposed to information about food waste were more likely to exhibit behaviors of avoiding food waste. This relationship was statistically significant with a *p*-value < 0.05. This shows that such communication programs are effective, and it is important for public authorities to make efforts to communicate on this issue or to support social initiatives in this direction.

For a significant impact on the environment and in the area of social responsibility related to FW reduction, a comprehensive approach is needed, which integrates all stages of the food supply chain, consumer education, effective collaboration between various civil society organizations and the private sector [72], implementation of innovative and sustainable production and consumption practices [35,60,73,74,75], sustainable energy production [76], expansion of national food bank branches [77], fueling innovations (technological, organizational, or marketing) [78], application of blockchain technology [79], the conversion of agri-food waste into innovative bioproducts [80,81], and the use of food waste in animal feed [82]. As Biggi et al. (2024) show, corporate social responsibility and legislative measures can have a synergistic effect on environmental management and FW reduction [83]. However, the need to create a societal culture to avoid and reduce FW was also reported internationally [84].

Environmental and social responsibility exhibit stronger positive associations with food waste avoidance, emphasizing the impact of individuals’ values on their waste reduction behaviors. Household discussions about food waste and exposure to information campaigns enhance food waste avoidance tendencies.

An overview on the variables that do not significantly influence the DV can be also informative. At the sample level, there were no statistically significant differences between men and women regarding the reporting of certain behaviors associated with FW. Similarly, with respect to age groups, the findings were not markedly different, using the reference category of the youngest individuals in the sample (20 years or below). Perhaps more surprisingly, the completion of tertiary education programs did not significantly influence FW behaviors. This may be attributed to the fact that such subjects are not covered in the university curriculum in Romania, and extracurricular programs of this nature aimed at students are lacking. The number of individuals in the household did not statistically impact the value of the FW index. Furthermore, income levels did not have a statistically significant relationship with the FW index. The lack of statistical significance in some relationships within this context may be influenced by the nuances already captured by the previously presented attitudinal-value model. The absence of a clear demographic and socio-economic profile regarding FW may also be relevant because, in Romania, there has not yet been sufficient time for such behaviors to crystallize solely within certain segments of the population. It is more likely that multiple directions of influence exist at both generational and socio-economic levels. For instance, some behaviors may still bear the imprint of the significant food shortages experienced during the Communist period of the country or the consumerism developed during the transition to a market economy since 1989. Nevertheless, in the absence of representative national studies, our research opens important niches for further investigation.

The issue of FW is complex and requires multiple and timely interventions along the food supply chain [85,86]. In the final stage of the chain, information campaigns and the need to educate household consumers, especially young people regarding the need to avoid and reduce FW is highlighted in numerous research papers [3,46,87]. Food literacy is an important determinant of dietary behavior [86]. Education in this direction must be carried out in the context of the three pillars of sustainability (social, economic, and environmental), within each generation and between generations, emphasizing an understanding of the resources involved in food production [87].

## 4. Conclusions

The present paper is an exploratory study on the behavior of domestic consumers in Romania regarding FW. The results have determined a composite index that measures concrete actions to avoid FW. The determined composite index assesses self-reported food waste avoidance behaviors at the individual level. A concept or construct that cannot be directly measured was thus represented. The model was tested using the stepwise linear regression model. According to the resulting model, the main predictors that determine concrete actions to avoid FW are the desire to reduce the negative impact on the environment; discussions about FW held at the household level and ways to reduce it; household food routines, especially regarding cooking at home; social circle; ecological and social responsibility of individuals; and awareness campaigns regarding the environmental, social, and economic impact of FW, carried out in all stages of the agri-food chain.

The social values and personal beliefs formed through an appropriate education for each generation can be used to promote a culture of avoiding food waste, which must be created and consolidated in Romania. A national culture of avoiding FW and sustainable FW management is essential in sustainable development, the current need for action is to mitigate the effects of climate change and ensure food safety and security.

Informing and educating conscious and responsible generations is the key to tackling this problem. Education and awareness efforts with the support of all stakeholders, followed by FW monitoring, are essential to changing attitudes and behaviors regarding this issue. Public awareness campaigns, school programs, and community initiatives can help promote a culture of less wasteful food consumption. In this context, the recommendations for politicians and government institutions refer to the need for education and awareness efforts, which are essential to change attitudes and behaviors regarding FW in Romania.

The research has some limitations related to the number of respondents. The findings offer a comprehensive image of the current FW behavior based on an online survey and a self-administered questionnaire, which does not necessarily represent the entire population of Romania.

Further studies are necessary regarding the Romanian consumer behavior related to FW, to obtain a general picture of this behavior at the national level. The research will be extended to the other links of the food supply chain (producers, processors, traders, and HoReCa).

## Figures and Tables

**Table 1:** Data of respondents.

Specification		Frequency	%	Specification		Frequency	%
Gender	Male	121	32.79	Age groups (years)	18–20	99	26.8
	Female	248	67.21		21–30	140	37.9
	Total	369	100		31–40	42	11.4
Domicile	Urban	253	68.56		41–50	56	15.2
	Rural	116	31.44		Above 50	32	8.7
	Total	369	100		Total	369	100

Source: Authors ‘own calculation.

**Table 2:** Level of education, income, and number of people in the respondents’ household.

Specification		Frequency	%	Specification		Frequency	%
The highest level of education completed	Education level below tertiary education	199	53.9%	Monthly expenditure of the family for the purchase of food *	Under 1000 lei	86	23.30
	Tertiary education level completed	170	46.1%		1001–2000 lei	182	49.32
					2001–3000 lei	75	20.33
					Above 3001 lei	26	7.05
					Total	369	100
	Total	369	100	Persons in Household	1	15	4.06
Family income *	Under 2500 lei	15	4.06		2	85	23.04
	2501–5000 lei	91	24.67		3	93	25.20
	5001–7500 lei	100	27.10		4	111	30.08
	7501–10,000 lei	85	23.04		5	25	6.78
	Above 10,001 lei	78	21.13		Above 6	40	10.84
	Total	369	100		Total	369	100

* 1 euro = 4.98 lei on 1 June 2024. Source: Authors’ own calculation.

**Table 3:** List of indicators included in the composite index for measuring FW avoidance (ranked by the mean value recorded at the sample level).

Behaviors	Mean	Std. Dev.	Variance	Coefficient of Variation (%)
i1 Proper food storage	5.58	1.49	2.21	26.71
i2 Reading food expiration dates carefully and consuming them on time	5.18	1.61	2.60	31.05
i3 Checking and prioritizing the use of food based on expiration dates	4.96	1.65	2.72	33.24
i4 Making a shopping list	4.72	1.76	3.08	37.34
i5 Cooking smaller portions	4.69	1.47	2.15	31.34
i6 Purchasing food in smaller quantities	4.66	1.54	2.35	32.98
i7 Reusing leftovers from the fridge	4.20	1.82	3.30	43.33
i8 Meal planning	4.18	1.68	2.81	40.18
i9 Purchasing discounted food nearing its expiration date	4.15	1.85	3.42	44.58
i10 Donating excess food to vulnerable individuals	3.89	1.72	2.97	44.19

Source: Authors’ own elaboration.

**Table 4:** List of independent variables.

Indicators	Mean (M)	Std. Dev. (SD)	Min	Max
The individual usually cooks at home (scale 1 to 5)	4.30	0.86	1	5
Reducing the negative environmental impact is an important motivation for making an effort to reduce food waste (scale 1 to 5)	3.93	0.97	1	5
Taking social responsibility through food consumption to alleviate global food issues is an important motivation for making an effort to reduce food waste (scale 1 to 5)	3.97	1.01	1	5
Most of the important people in individual’s life disapprove of food being wasted (scale 1 to 5)	2.82	1.30	1	5
The individuals had discussions in their household about food waste and ways to reduce it (yes = 1)	0.83	0.37	0	1
The individuals heard about information campaigns or news that raise awareness about food waste and offer tips for reducing it (yes = 1)	0.62	0.49	0	1
Gender (male)	0.33	0.47	0	1
Age (number of years)	30.22	12.91	18	73

Source: Authors’ own elaboration.

**Table 5:** Results of the stepwise linear regression model: DV—The composite index for measuring food waste avoidance.

		Standardized Coefficients (Beta)					
	Models	M1	M2	M3	M4	M5	M6
Predictors							
IV1. Reducing the negative environmental impact is an important motivation for making an effort to reduce food waste		0.432 ***	0.416 ***	0.414 ***	0.425 ***	0.302 ***	0.304 ***
IV2. The individuals had discussions in their household about food waste and ways to reduce it			0.198 ***	0.184 ***	0.182 ***	0.171 ***	0.157 **
IV3. The individual usually cooks at home				0.138 **	0.138 **	0.129 **	0.122 **
IV4. Most of the important people in an individual’s life disapprove of food being wasted					−0.123 **	-0.125 **	-0.126 **
IV5. Taking social responsibility through food consumption to alleviate global food issues is an important motivation for making an effort to reduce food waste						0.169 *	0.170 *
IV6. The individuals heard about information campaigns or news that raise awareness about food waste and offer tips for reducing it							0.102 *
Adjusted R Square		0.184	0.221	0.238	0.251	0.262	0.270

Notes: * *p* < 0.05; ** *p* < 0.01; *** *p* < 0.001 (please see Supplementary Materials File S2 for the complete results of the stepwise linear regression analysis). Source: Authors’ own elaboration.

## Data Availability

The original contributions presented in the study are included in the article/supplementary material, further inquiries can be directed to the corresponding author.

## Supplementary Materials

The following supporting information can be downloaded at: https://www.mdpi.com/article/10.3390/foods13203313/s1, File S1: Factor analysis-FW index, File S2: Stepwise regression model.
