# Supplementary material for: Tackling Food Waste: An Exploratory Case Study on Consumer Behavior in Romania
Source: Foods. 2024 Oct 18;13(20):3313. doi: 10.3390/foods13203313 (PMC11507217; doi:10.3390/foods13203313)
Supplement: Supplementary file 1 [file foods-13-03313-s001.zip › Supplementary File S1 - Factor Analysis - FW index.pdf]

File S1. Results of the Factor Analysis conducted with the items included in the summative FW index – Extraction Method: Principal Component Analysis

KMO and Bartlett's Test		
Kaiser-Meyer-Olkin Measure of Sampling Adequacy.		.905
Bartlett's Test of Sphericity	Approx. Chi-Square	1642.797
	df	45
	Sig.	0.000

Communalities

	Initial	Extraction
Reusing leftovers from the fridge	1.000	.457
Cooking smaller portions	1.000	.599
Meal planning	1.000	.459
Making a shopping list	1.000	.446
Checking and prioritizing the use of food based on expiration dates	1.000	.642
Proper food storage	1.000	.631
Donating excess food to vulnerable individuals	1.000	.291
Reading food expiration dates carefully and consuming them on time	1.000	.627
Purchasing food in smaller quantities	1.000	.564
Purchasing discounted food nearing its expiration date	1.000	.271

Extraction Method: Principal Component Analysis.

Total Variance Explained

Component	Initial Eigenvalues			Extraction Sums of Squared Loadings		
	Total	% of Variance	Cumulative %	Total	% of Variance	Cumulative %
1	4.988	49.883	49.883	4.988	49.883	49.883
2	.919	9.187	59.070			
3	.809	8.094	67.165			
4	.767	7.667	74.832			
5	.619	6.189	81.021			
6	.513	5.133	86.154			
7	.420	4.203	90.357			
8	.377	3.771	94.128			
9	.348	3.484	97.612			
10	.239	2.388	100.000			

Extraction Method: Principal Component Analysis.

Component Matrix[a]

	Component
	1
Reusing leftovers from the fridge	.676
Cooking smaller portions	.774
Meal planning	.677
Making a shopping list	.668
Checking and prioritizing the use of food based on expiration dates	.802
Proper food storage	.795
Donating excess food to vulnerable individuals	.540
Reading food expiration dates carefully and consuming them on time	.792
Purchasing food in smaller quantities	.751
Purchasing discounted food nearing its expiration date	.521

Extraction Method: Principal Component Analysis.

a. 1 components extracted.

Correlation Matrix

	Reusing leftovers from the fridge	Cooking smaller portions	Meal planning	Making a shopping list	Checking and prioritizing the use of food based on expiration dates	Proper food storage	Donating excess food to vulnerable individuals	Reading food expiration dates carefully and consuming them on time
Reusing leftovers from the fridge	1.000	.563***	.444***	.451***	.454***	.451***	.298***	.408***
Cooking smaller portions	.563***	1.000	.503***	.466***	.533***	.573***	.338***	.532***
Meal planning	.444***	.503***	1.000	.552***	.432***	.419***	.351***	.389***
Making a shopping list	.451***	.466***	.552***	1.000	.482***	.436***	.231***	.392***
Checking and prioritizing the use of food based on expiration dates	.454***	.533***	.432***	.482***	1.000	.646***	.408***	.727***
Proper food storage	.451***	.573***	.419***	.436***	.646***	1.000	.355***	.670***
Donating excess food to vulnerable individuals	.298***	.338***	.351***	.231***	.408***	.355***	1.000	.373***
Reading food expiration dates carefully and consuming them on time	.408***	.532***	.389***	.392***	.727***	.670***	.373***	1.000
Purchasing food in smaller quantities	.384***	.539***	.420***	.416***	.512***	.570***	.347***	.613***
Purchasing discounted food nearing its expiration date	.310***	.315***	.291***	.277***	.326***	.340***	.253***	.322***

Notes: *$p < 0.05$; **$p < 0.01$; ***$p < 0.001$
