# Supplementary material for: Tackling Food Waste: An Exploratory Case Study on Consumer Behavior in Romania
Source: Foods. 2024 Oct 18;13(20):3313. doi: 10.3390/foods13203313 (PMC11507217; doi:10.3390/foods13203313)
Supplement: Supplementary file 1 [file foods-13-03313-s001.zip › Supplementary File S2 - Stepwise regression model.pdf]

File S2. Results of the stepwise linear regression model:
DV – The composite index for measuring food waste avoidance

Model	Coefficients	Unstandardized Coefficients		Standardized Coefficients	t	*p*
		B	Std. Err.	Beta		
1	(Constant)	2.588	0.229		11.286	0.000
	Reducing the negative environmental impact is an important motivation for making an effort to reduce food waste	0.517	0.057	0.432	9.139	0.000
Adjusted R Square 0,184						
2	(Constant)	2.153	0.246		8.754	0.000
	Reducing the negative environmental impact is an important motivation for making an effort to reduce food waste	0.498	0.055	0.416	8.993	0.000
	The individuals had discussions in their household about food waste and ways to reduce it	0.611	0.142	0.198	4.288	0.000
Adjusted R Square 0,221						
3	(Constant)	1.404	0.349		4.025	0.000
	Reducing the negative environmental impact is an important motivation for making an effort to reduce food waste	0.496	0.055	0.414	9.050	0.000
	The individuals had discussions in their household about food waste and ways to reduce it	0.567	0.142	0.184	4.000	0.000
	The individual usually cooks at home	0.185	0.062	0.138	3.001	0.003
Adjusted R Square 0,238						
4	(Constant)	1.661	0.359		4.632	0.000
	Reducing the negative environmental impact is an important motivation for making an effort to reduce food waste	0.509	0.055	0.425	9.334	0.000
	The individuals had discussions in their household about food waste and ways to reduce it	0.561	0.140	0.182	3.995	0.000
	The individual usually cooks at home	0.185	0.061	0.138	3.032	0.003
	Most of the important people in an individual's life disapprove of food being wasted	-0.109	0.040	-0.123	-2.705	0.007
Adjusted R Square 0,251						
5	(Constant)	1.552	0.358		4.330	0.000
	Reducing the negative environmental impact is an important motivation for making an effort to reduce food waste	0.362	0.080	0.302	4.546	0.000

	The individuals had discussions in their household about food waste and ways to reduce it	0.526	0.140	0.171	3.755	0.000
	The individual usually cooks at home	0.173	0.061	0.129	2.845	0.005
	Most of the important people in an individual's life disapprove of food being wasted	-0.110	0.040	-0.125	-2.763	0.006
	Taking social responsibility through food consumption to alleviate global food issues is an important motivation for making an effort to reduce food waste	0.195	0.077	0.169	2.536	0.012
Adjusted R Square 0,262						
6	(Constant)	1.470	0.358		4.103	0.000
	Reducing the negative environmental impact is an important motivation for making an effort to reduce food waste	0.365	0.079	0.304	4.609	0.000
	The individuals had discussions in their household about food waste and ways to reduce it	0.485	0.141	0.157	3.449	0.001
	The individual usually cooks at home	0.163	0.061	0.122	2.694	0.007
	Most of the important people in an individual's life disapprove of food being wasted	-0.112	0.040	-0.126	-2.817	0.005
	Taking social responsibility through food consumption to alleviate global food issues is an important motivation for making an effort to reduce food waste	0.195	0.076	0.170	2.556	0.011
	The individuals heard about information campaigns or news that raise awareness about food waste and offer tips for reducing it	0.241	0.107	0.102	2.251	0.025
Adjusted R Square 0,270						

Excluded Variables from the stepwise linear regression model: DV – The composite index for measuring food waste avoidance

Model		Beta In	t	Sig.	Partial Correlation	Collinearity Statistics (Tolerance)
1	The individual usually cooks at home	.157	3.366	0.001	0.174	1.000
	The individuals heard about information campaigns or news that raise awareness about food waste and offer tips for reducing it	.134	2.865	0.004	0.148	1.000

	The individuals had discussions in their household about food waste and ways to reduce it	.198	4.288	0.000	0.219	0.994
	Most of the important people in an individual's life disapprove of food being wasted	-.125	-2.668	0.008	-0.139	0.992
	Taking social responsibility through food consumption to alleviate global food issues is an important motivation for making an effort to reduce food waste	.211	3.061	0.002	0.158	0.460
	Male (reference category: Female)	-.004	-0.084	0.933	-0.004	0.970
	Age 21-30 (reference category 20 years or below)	-.055	-1.157	0.248	-0.061	0.994
	Age 31-40 (reference category 20 years or below)	-.026	-0.542	0.588	-0.028	0.998
	Age 41-50 (reference category 20 years or below)	.066	1.391	0.165	0.073	0.987
	Age over 50 (reference category 20 years or below)	.095	2.027	0.043	0.106	1.000
	Tertiary education completed (reference category: No tertiary education completed)	.050	1.054	0.293	0.055	1.000
	Number of persons in the household	.007	0.147	0.883	0.008	1.000
	Earnings (scale from 1 to 5)	.084	1.794	0.074	0.094	1.000
2	The individual usually cooks at home	.138	3.001	0.003	0.156	0.989
	The individuals heard about information campaigns or news that raise awareness about food waste and offer tips for reducing it	.109	2.346	0.020	0.122	0.981
	Most of the important people in an individual's life disapprove of food being wasted	-.123	-2.669	0.008	-0.139	0.992
	Taking social responsibility through food consumption to alleviate global food issues is an important motivation for making an effort to reduce food waste	.182	2.677	0.008	0.139	0.454
	Male (reference category: Female)	.011	0.231	0.818	0.012	0.965
	Age 21-30 (reference category 20 years or below)	-.030	-0.651	0.516	-0.034	0.979

	Age 31-40 (reference category 20 years or below)	-.021	-0.453	0.651	-0.024	0.998
	Age 41-50 (reference category 20 years or below)	.042	0.901	0.368	0.047	0.972
	Age over 50 (reference category 20 years or below)	.078	1.696	0.091	0.089	0.992
	Tertiary education completed (reference category: No tertiary education completed)	.040	0.861	0.390	0.045	0.997
	Number of persons in the household	.005	0.104	0.917	0.005	1.000
	Earnings (scale from 1 to 5)	.068	1.481	0.140	0.077	0.993
3	The individuals heard about information campaigns or news that raise awareness about food waste and offer tips for reducing it	.099	2.160	0.031	0.113	0.976
	Most of the important people in an individual's life disapprove of food being wasted	-.123	-2.705	0.007	-0.141	0.992
	Taking social responsibility through food consumption to alleviate global food issues is an important motivation for making an effort to reduce food waste	.167	2.472	0.014	0.129	0.452
	Male (reference category: Female)	.037	0.791	0.429	0.042	0.933
	Age 21-30 (reference category 20 years or below)	-.029	-0.623	0.534	-0.033	0.979
	Age 31-40 (reference category 20 years or below)	-.007	-0.148	0.882	-0.008	0.987
	Age 41-50 (reference category 20 years or below)	.032	0.679	0.498	0.036	0.966
	Age over 50 (reference category 20 years or below)	.065	1.422	0.156	0.075	0.982
	Tertiary education completed (reference category: No tertiary education completed)	.035	0.775	0.439	0.041	0.996
	Number of persons in the household	-.018	-0.398	0.691	-0.021	0.972
	Earnings (scale from 1 to 5)	.064	1.400	0.162	0.073	0.992
4	The individuals heard about information campaigns or news that raise awareness about food waste and offer tips for reducing it	.101	2.227	0.027	0.116	0.975

	Taking social responsibility through food consumption to alleviate global food issues is an important motivation for making an effort to reduce food waste	.169	2.536	0.012	0.132	0.451
	Male (reference category: Female)	.028	0.592	0.554	0.031	0.928
	Age 21-30 (reference category 20 years or below)	-.026	-0.565	0.572	-0.030	0.978
	Age 31-40 (reference category 20 years or below)	-.015	-0.328	0.743	-0.017	0.983
	Age 41-50 (reference category 20 years or below)	.029	0.633	0.527	0.033	0.966
	Age over 50 (reference category 20 years or below)	.067	1.478	0.140	0.078	0.982
	Tertiary education completed (reference category: No tertiary education completed)	.033	0.734	0.464	0.039	0.996
	Number of persons in the household	-.001	-0.026	0.979	-0.001	0.953
	Earnings (scale from 1 to 5)	.059	1.305	0.193	0.069	0.990
5	The individuals heard about information campaigns or news that raise awareness about food waste and offer tips for reducing it	.102	2.251	0.025	0.118	0.975
	Male (reference category: Female)	.034	0.734	0.463	0.039	0.925
	Age 21-30 (reference category 20 years or below)	-.023	-0.507	0.613	-0.027	0.978
	Age 31-40 (reference category 20 years or below)	-.020	-0.437	0.663	-0.023	0.981
	Age 41-50 (reference category 20 years or below)	.029	0.626	0.532	0.033	0.966
	Age over 50 (reference category 20 years or below)	.069	1.519	0.130	0.080	0.982
	Tertiary education completed (reference category: No tertiary education completed)	.033	0.726	0.468	0.038	0.996
	Number of persons in the household	-.003	-0.074	0.941	-0.004	0.953
	Earnings (scale from 1 to 5)	.065	1.449	0.148	0.076	0.988
6	Male (reference category: Female)	.026	0.563	0.574	0.030	0.919
	Age 21-30 (reference category 20 years or below)	-.024	-0.522	0.602	-0.028	0.978
	Age 31-40 (reference category 20 years or below)	-.007	-0.151	0.880	-0.008	0.965
	Age 41-50 (reference category 20 years or below)	.027	0.586	0.558	0.031	0.966

	Age over 50 (reference category 20 years or below)	.057	1.248	0.213	0.066	0.966
	Tertiary education completed (reference category: No tertiary education completed)	.029	0.650	0.516	0.034	0.995
	Number of persons in the household	-.005	-0.103	0.918	-0.005	0.953
	Earnings (scale from 1 to 5)	.064	1.417	0.157	0.075	0.987

ANOVA for the stepwise linear regression model:<br>DV – The composite index for measuring food waste avoidance						
Model		Sum of Squares	df	Mean Square	F	Sig.
1	Regression	90.958	1	90.958	83.514	.000
	Residual	397.536	365	1.089		
	Total	488.494	366			
2	Regression	110.075	2	55.037	52.940	.000
	Residual	378.419	364	1.040		
	Total	488.494	366			
3	Regression	119.238	3	39.746	39.073	.000
	Residual	369.256	363	1.017		
	Total	488.494	366			
4	Regression	126.552	4	31.638	31.643	.000
	Residual	361.942	362	1.000		
	Total	488.494	366			
5	Regression	132.886	5	26.577	26.980	.000
	Residual	355.609	361	.985		
	Total	488.494	366			
6	Regression	137.821	6	22.970	23.581	.000
	Residual	350.673	360	.974		
	Total	488.494	366			
